# H3K9 and H3K14 acetylation co-occur at many gene regulatory elements, while H3K14ac marks a subset of inactive inducible promoters in mouse embryonic stem cells

**DOI:** 10.1186/1471-2164-13-424

**Published:** 2012-08-24

**Authors:** Krishanpal Karmodiya, Arnaud R Krebs, Mustapha Oulad-Abdelghani, Hiroshi Kimura, Laszlo Tora

**Affiliations:** 1 Institut de Génétique et de Biologie Moléculaire et Cellulaire (IGBMC), CNRS UMR 7104, INSERM U 964, Université de Strasbourg, BP 10142-67404 ILLKIRCH Cedex, CU de Strasbourg, France; 2 Graduate School of Frontier Biosciences, Osaka University, 1-3 Yamadaoka, Suita, Osaka, 565-0871, Japan; 3 Present address: Friedrich Miescher Institute for Biomedical Research, 4058, Basel, Switzerland

**Keywords:** ChIP-seq, Histone acetylation, CpG islands, Embryonic stem cells, Gene regulation, Genome-wide mapping, Bivalent promoters, Epigenetics

## Abstract

**Background:**

Transcription regulation in pluripotent embryonic stem (ES) cells is a complex process that involves multitude of regulatory layers, one of which is post-translational modification of histones. Acetylation of specific lysine residues of histones plays a key role in regulating gene expression.

**Results:**

Here we have investigated the genome-wide occurrence of two histone marks, acetylation of histone H3K9 and K14 (H3K9ac and H3K14ac), in mouse embryonic stem (mES) cells. Genome-wide H3K9ac and H3K14ac show very high correlation between each other as well as with other histone marks (such as H3K4me3) suggesting a coordinated regulation of active histone marks. Moreover, the levels of H3K9ac and H3K14ac directly correlate with the CpG content of the promoters attesting the importance of sequences underlying the specifically modified nucleosomes. Our data provide evidence that H3K9ac and H3K14ac are also present over the previously described bivalent promoters, along with H3K4me3 and H3K27me3. Furthermore, like H3K27ac, H3K9ac and H3K14ac can also differentiate active enhancers from inactive ones. Although, H3K9ac and H3K14ac, a hallmark of gene activation exhibit remarkable correlation over active and bivalent promoters as well as distal regulatory elements, a subset of inactive promoters is selectively enriched for H3K14ac.

**Conclusions:**

Our study suggests that chromatin modifications, such as H3K9ac and H3K14ac, are part of the active promoter state, are present over bivalent promoters and active enhancers and that the extent of H3K9 and H3K14 acetylation could be driven by cis regulatory elements such as CpG content at promoters. Our study also suggests that a subset of inactive promoters is selectively and specifically enriched for H3K14ac. This observation suggests that histone acetyl transferases (HATs) prime inactive genes by H3K14ac for stimuli dependent activation. In conclusion our study demonstrates a wider role for H3K9ac and H3K14ac in gene regulation than originally thought.

## Background

Embryonic stem (ES) cells provide an important model system to study developmental regulation and hold significant potential for clinical therapeutics because of their unique capabilities to self re-new and differentiate into multiple lineages (reviewed in [1]). The chromatin of pluripotent ES cells have unique characteristics, including an open conformation, a hyper-dynamic organization of chromatin proteins, and less condensed heterochromatin domains, suggesting the plasticity of the genome in ES cells (reviewed in [2]).

Different modifications of chromatin are associated with variable functions. Histone modifications such as trimethylation of H3 lysine 4 (H3K4me3) and hyperacetylation of histone H3 and H4 are known as active marks and are often associated with ongoing transcription [3,4]. On the other hand, methylation of H3K9 and H3K27, are known as repressive marks and are associated with gene silencing [5]. Promoters of key regulatory genes have unique chromatin modification signatures, which contain both an active histone mark, H3K4me3, as well as a repressive histone mark, H3K27me3, also known as bivalent promoters, and are thought to be poised for gene activation during differentiation [6-8]. Distal regulatory regions, such as enhancers, are enriched in H3K4me1 (as compared to H3K4me3), histone acetyl transferase (HAT) co activators (i.e. p300 or ATAC) and have an open chromatin structure [4,9,10]. Not only promoters, but enhancer regions were also shown to be poised for gene activation during differentiation as only active enhancers are marked by the H3K27ac modification [11,12].

One of the most studied modifications of histones is acetylation of specific lysine (K) residues, which generally correlates with gene activation. The level of histone acetylation is regulated by the activity of both histone acetyl transferases (HATs) and histone deacetylases (HDACs), which acetlylate and deacetylate lysine residues of the N terminal histone tails, respectively. Genetic and biochemical studies suggested that HATs have rather specific roles in gene activation, while genome-wide experiments rather suggested that HATs are often recruited simultaneously, and together are acetylating multiple lysine residues at a given loci [13,14]. Thus, the biological function of histone acetylation may be rather additive than specific. In ES cells, acetylation of H3K9 was shown to predict the pluripotency and reprogramming capacity [15] and its level reduces with ES cell differentiation [16]. Recent genome-wide studies shed light on various histone modifications in mES cells [6], however, the genome-wide role of histone H3 acetylation in mES cells is poorly understood. In this study, we have investigated two histone acetylation marks, H3K9 and H3K14. Acetylation of H3K9 is mainly performed by histone acetyl transferases GCN5/PCAF and/or Tip60, whereas acetylation of H3K14 is mediated by GCN5/PCAF, p300/CBP and/or Myst3 [17-19].

The lack of in-depth studies concerning the role of H3K14ac was due to the absence of reliable and specific antibodies. Antibodies against modified histone tails are central research tools in studying chromatin biology at a genome-wide level. Thus, we have developed a new specific ChIP-grade antibody against the H3K14 acetylation mark. By using the antibody we have developed against H3K14ac and another published commercial antibody against H3K9ac, we made genome-wide location analysis of these two acetylation marks in mouse (m) ES cells, and compared the presence of two marks over various genomic regions. Our study suggests that these two marks are present not only over promoters of actively transcribed genes, but also on the developmentally regulated bivalent promoters, as well as over active enhancers in mES cells. Moreover, the degree of H3K9 and H3K14 acetylation correlates with the CpG content of the promoters and transcription level of the genes. Finally, we observed differential presence of these two acetylation marks over a subset of inactive genes, which is marked by low level of H3K14ac and thus seemed to be prepared for future activation.

## Results

### Genome-wide acetylation profiles of H3K9 and H3K14 correlate with each other

To understand the role of histone H3 acetylation at positions K9 and K14 in mouse ES cells, we have systematically tested the available antibodies raised against these modified histone tails. We and others have found that the anti-H3K9ac antibody from Abcam (ab4441) is specific for the corresponding modification in various applications including chromatin immunoprecipitation (ChIP) [20,21]. However, the anti-H3K14ac antibody from Upstate (07-353), which was used earlier for genome-wide localization of H3K14ac [3], was shown to cross-react with other histone modifications [20] and was found non-applicable for chromatin immunoprecipitation coupled high throughput sequencing (ChIP-seq) [21]. Our ELISA tests showed that it not only cross-reacts with with the H4K5acK12ac peptide, it also recognizes the non--acetylated H3K14 peptide ( Additional file 1: Figure S1). To overcome this limitation and to study the real genome-wide distribution of H3K14ac, we raised a specific mouse monoclonal antibody (mAb) against this modification and confirmed its specificity in several different tests ( Additional file 2: Figure S2).

To gain insight in the genome-wide acetylation profile of H3K9 and H3K14 residues, ChIP-seq was performed using the commercially available antibody against H3K9ac and the new antibody developed against H3K14ac in this study. Peaks of local enrichment for H3K9ac and H3K14ac were determined after sequence alignment and normalization to input DNA. Further to validate the peaks obtained in ChIP-seqs for H3K9ac (using ab4441 antibody) and H3K14ac (using 13HH3-1A5 antibody), we performed ChIP-qPCR on randomly selected genomic loci enriched for H3K9ac and H3K14ac. All the selected peaks of H3K9 and H3K14 acetylation from the ChIP-seq experiments were validated by ChIP-qPCR ( Additional file 3: Figure S3 and Additional file 4: Table S1.

**Figure 1:**
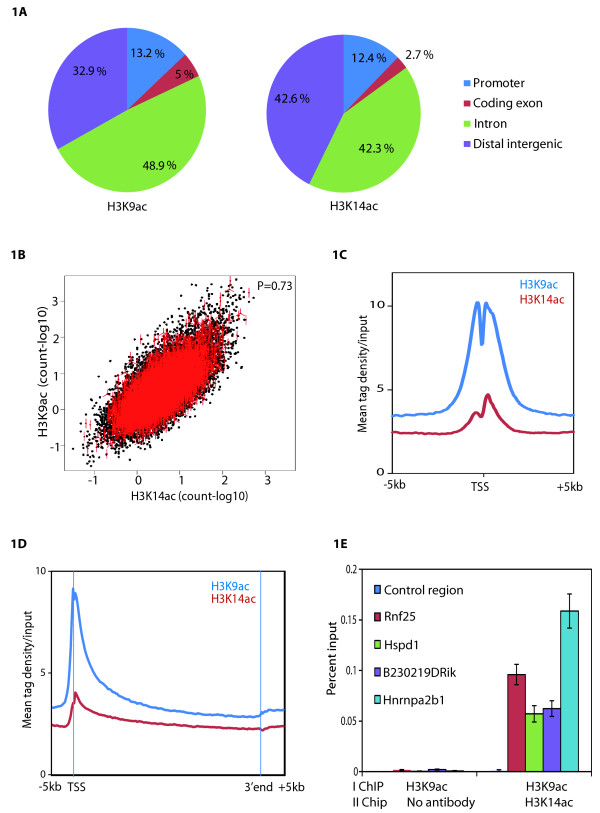
**Genomic distribution of H3K9ac and H3K14ac in mES cells. **(**A**) Distribution of H3K9ac and H3K14ac peaks over the promoters (2000 bp upstream of TSS,), coding exons, introns and distal intergenic regions. Many of the H3K9 and H3K14 acetylation peaks are at distal intergenic regions. (**B**) Dot plot representation of genome-wide co-localization analysis of the H3K9ac and H3K14ac modifications over the 15595 combined promoter list of H3K9 and H3K14ac suggests a strong correlation between these two modifications at promoters. (**C**) Average input normalized profile of 15595 combined promoter list of H3K9 and H3K14ac around the transcription starts sites shows bimodal distribution. (**D**) Average input normalized whole gene profiles for H3K9ac and H3K14ac modifications over 15595 combined promoter list of H3K9 and H3K14ac. (**E**) Sequential ChIP–qPCR quantification for co-occupancy of H3K9ac (primary ChIP) and H3K14ac (secondary ChIP) at randomly selected H3K9 and H3K14 acetylated loci suggest that these loci are co-marked with H3K9 as well H3K14 acetylation. Enrichment after first ChIP using H3K9ac followed by re-ChIP with no antibody was used as a control. Primer sequences used in ChIP-qPCR is provided in Additional file 4: Table S1. Error bars represent the standard deviation for three technical replicates.

In order to test whether H3K9ac and H3K14ac modifications have differential preference over various chromatin regions, we compared their presence over promoters (2000 bp upstream of transcription start sites (TSSs)), coding exons, introns and distal intergeneic regions (Figure 1A), which represent 2.5%, 1.6%, 38.7% and 57.2% of the total genome, respectively [22]. First, peaks of H3K9ac and H3K14ac local enrichment were determined after sequence alignment and normalization to input DNA using MACS [23]. Our analyses show that H3K9ac and H3K14ac peaks are distributed over all four genomic regions and the frequency of distribution over promoters is 13.2% and 12.4%, respectively (Figure 1A). However, approximately 85% of both the H3K9ac and H3K14ac marks are observed in distal intergenic and intronic regions with significant enrichments, comparable to promoters ( Additional file 5: Figure S4), suggesting that these two modifications may have a role at distal intergeneic and intronic regions. Further to compare H3K9 and H3K14ac marks, a combined list of binding sites at transcription start sites (TSSs) was established containing 15595 TSSs. The comparison of H3K9ac and H3K14ac over these TSSs shows a Pearson correlation coefficient of 0.73 (Figure 1B), suggesting that the studied two H3 acetylation marks are present simultaneously on promoters (Figure 1C). Moreover, H3K9 and H3K14 acetylations have a characteristic bimodal distribution around the TSSs, with one peak upstream of the TSS, another single peak (stronger in the case of H3K14ac) downstream of the TSS, and depletion of the signal right on the TSS (Figure 1C). To examine the distribution of these two histone marks over the gene body, composite profile of both marks spanning the entire gene body and extending 5 kb upstream from TSSs and 5 kb downstream of the 3’ end of the genes for combined list of binding sites over TSSs (15595) was generated (Figure 1D). The H3K9ac and H3K14ac distribution profiles around TSSs suggest that both marks are predominantly located in regions surrounding the TSSs of genes. Further to confirm that the co-occupancy of H3K9 and H3K14 observed is not because of cellular heterogeneity, we performed sequential ChIP for H3K9ac followed by H3K14ac (Figure 1E). Sequential ChIP demonstrates that genomic loci are acetylated simultaneously both at H3K9 as well H3K14. Thus, our analyses suggest that on the genome H3K9ac and H3K14ac are mostly present at distal intergenic and intronic regions, specifically enriched at promoters when localized in the vicinity of genes and that the two acetylation marks co-occur at promoters as well as other locations. 

### Levels of H3K9 and H3K14 acetylations correlate with the magnitude of gene expression

**Figure 2:**
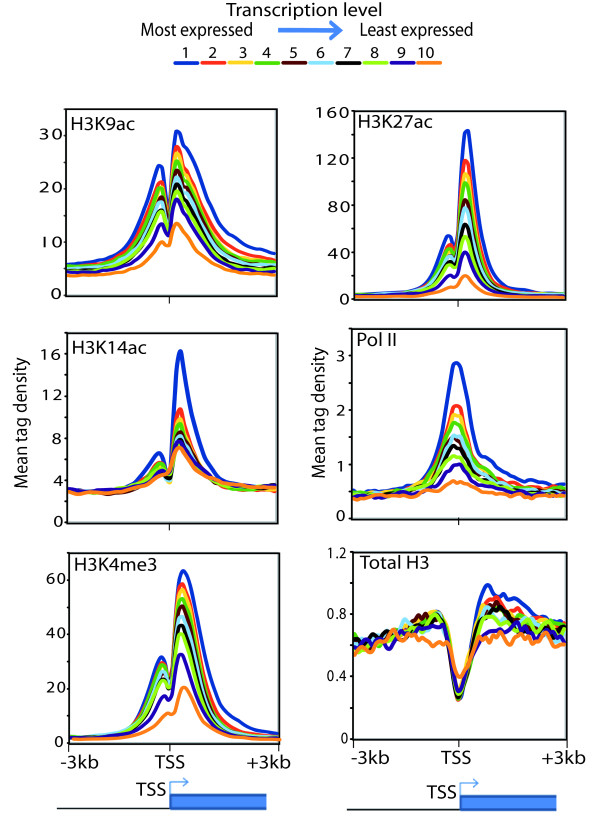
**Level of H3K9 and H3K14 acetylation correlates with magnitude of gene expression. **A total of 12000 expressed genes in mES cells were divided into ten groups based on their expression levels, from the top 10% (blue, group 1) to the lowest 10% (purple, group 10). Mean tag densities of active histone marks; H3K9ac, H3K14ac, H3K4me3, H3K27ac and Pol II within (-/+) 3 kb are positively correlated with the transcription level of the genes. On the other hand, total H3 densities in the same regions are negatively correlated with the transcription level.

Global transcription is a hallmark of pluripotent ES cells that contributes to plasticity and lineage specification [24]. Histone modifications are known to act in a combinatorial fashion to determine the overall outcome of the gene expression [3]. To explore the correlative relationship between various active histone marks detected at promoters and the transcription of the corresponding genes in mES cells, we compared the level of various active histone marks with the transcriptional level of the genes. Densities of active histone marks (H3K9ac, H3K14ac, H3K4me3 and H3K27ac), as well as total H3 and RNA polymerase II (Pol II), within a 3000 bp window flanking the TSSs of the expressed genes (12100) were collected. All expressed genes [8] were divided into ten categories ranked on the basis of their expression level. Presence of various active histone marks was analyzed over these categories. Analysis of histone H3 occupancy, histone modifications (H3K9ac, H3K14ac, H3K4me3 and H3K27ac) and Pol II around the TSSs suggest that depletion of the total histone H3 signal and enrichment of the active promoter marks (H3K9ac, H3K14ac, H3K4me3 and H3K27ac) at or around TSSs correlate with the increase in gene expression levels. Interestingly, H3K9ac is more spread than the other analyzed active histone marks around the TSSs (Figure 2). While Pol II is enriched at or slightly downstream of the TSSs, on these sites the nucleosomes (H3) are depleted (Figure 2). This genome-wide observation with various active histone marks is consistent with the notion that H3K9ac and H3K4me3 near the TSSs destabilize interaction between histones and DNA leading to nucleosome eviction [25,26]. Taken together, these results suggest that level of active histone marks (H3K4me3, H3K9ac, H3K14ac and H3K27ac) over the active promoter chromatin state correlates with the magnitude of gene expression. 

### H3K9 and H3K14 acetylation levels correlate with the CpG content

**Figure 3:**
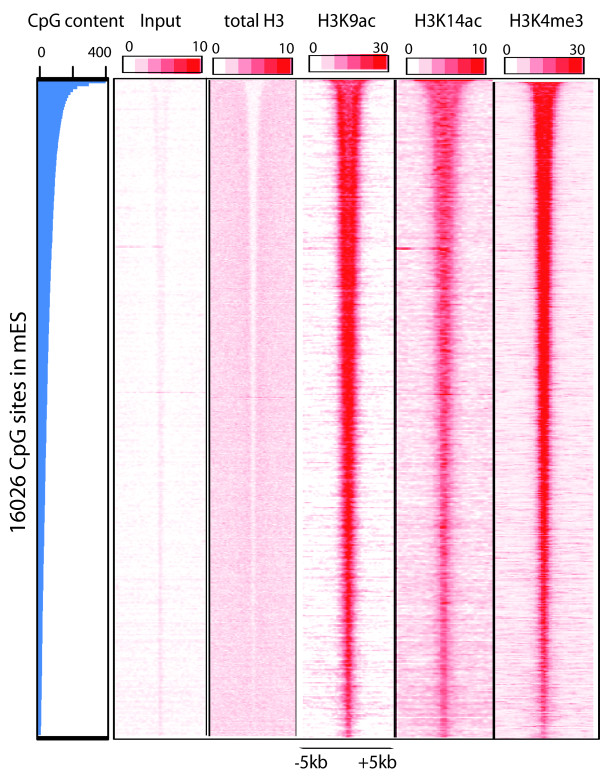
**Correlation between the CpG content and the H3K9ac and H3K14ac level. **16026 CpG island sites associated with genes were sorted in descending order (top to bottom) on the basis of CpG content and the total H3, H3K9ac, H3K14ac and H3K4me3 were examined over them. CpG content on these sites correlates with the level of H3K9 and H3K14 acetylation as well as with the H3K4me3.

Cytosine-phosphate diester-guanine (CpG) islands are usually found at the 5’ end of the regulatory regions of genes [27]. CpG islands are GC rich, predominantly non-methylated and their content correlates with H3K4me3 chromatin modification. To explore the relationship between the CpG content and the levels of H3K9ac and H3K14ac, we took all the CpG island sites (16026) from UCSC genome browser [28] and sorted them according to their CpG content. On these CpG islands, which are sorted on the basis of their increasing CpG content, we looked for the H3K9ac and H3K14ac profile. We found that indeed CpG content correlates with the level of H3K9 and H3K14 acetylation, as acetylation over those sites increases in parallel with the CpG content (Figure 3). This in turn suggests that the levels of H3K9 and H3K14 acetylations on the nucleosomes positioning around the TSSs of the promoters correlate with the CpG content of the underlying DNA sequence. This is in accordance with the fact that CpG enriched genes are generally housekeeping and are widely expressed [8,29]. 

### H3K9 and H3K14 acetylations occur at active enhancers

**Figure 4:**
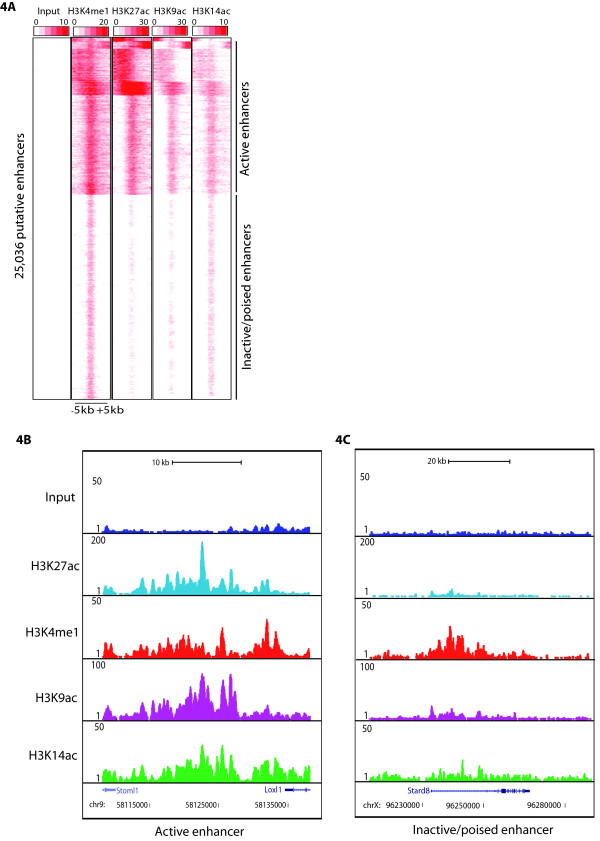
**H3K9ac and H3K14ac mark active enhancers along with H3K27ac. **(**A**) Heatmap of the signal density using k-means clustering observed on 25036 putative enhancers (-/+ 5 kb) from mES cells for H3K4me1 (mark of putative enhancers), H3K27ac, H3K9ac and H3K14ac. On the basis of presence or absence of H3K27ac enhancers are categorized as active or poised/inactive. UCSC genome browser track of representative examples of (**B**) active enhancer and (**C**) poised/inactive enhancer. Active enhancers have significant enrichment of H3K9ac and H3K14ac as compared to poised/inactive enhancers as was observed for H3K27ac.

Transcription from enhancers resulting in enhancer RNAs (eRNAs) play important regulatory role in maintenance of gene expression programs [30-32]. Enhancers are key cis-regulatory elements that can affect gene expression independent of their orientation or distance in a cell type specific manner [9]. Enhancers are marked by the presence of H3K4me1, DNase I hypersensitivity and histone acetyl transferases such as p300 [4,9] or the GCN5/PCAF-containing ATAC complex [10]. The important proportion of H3K9 and H3K14 acetylation sites in distal intergenic regions (Figure 1A) motivated us to further analyze these sites for the presence of various histone modifications, Pol II and p300 that are indicative of enhancers. Identification of H3K9ac and H3K14ac peaks in intergenic regions is described in Materials and Methods. During this analysis we found a strong correlation between H3K9ac or H3K14ac intergenic sites with either, H3K4me1, H3K27ac, the presence of Pol II and p300 suggesting that H3K9ac and H3K14ac mark also enhancers ( Additional file 6: Figure S5). To confirm the presence of H3K9ac and H3K14ac over enhancers, we took 25036 putative enhancers reported in mES cells [11] and subjected them to k-means clustering using seqMiner [33]. Further, H3K27ac was used to distinguish active and inactive/poised enhancers. Our analyses show that active enhancers are marked by the strong presence of H3K9ac and H3K14ac along with H3K27ac and H3K4me1 (Figure 4A and 4B) and those inactive/poised enhancers are marked by the presence of H3K4me1 together with relatively weak levels of H3K14ac (Figure 4A and 4C). Thus, our study suggests that H3K9ac and H3K14ac mark enhancers and can further discriminate active enhancers from poised/inactive enhancers. 

### Bivalent promoters are also marked by H3K9 and K14 acetylation in pluripotent mES cells

Many promoters of developmentally regulated genes in mES cells are marked by H3K4me3 (active histone mark) as well as polycomb mediated repressive histone mark, H3K27me3 [6,7] and are known as bivalent promoters. Recent studies have shown that these bivalent promoters are also bound by Pol II and are transcribed at very low level [34]. As these promoters have H3K4me3 and show a very low level of active transcription we looked for the presence of H3K9 and H3K14 acetylation over the bivalent promoters to see if this low level of transcription would also associated with acetylation on these promoters.

**Figure 5:**
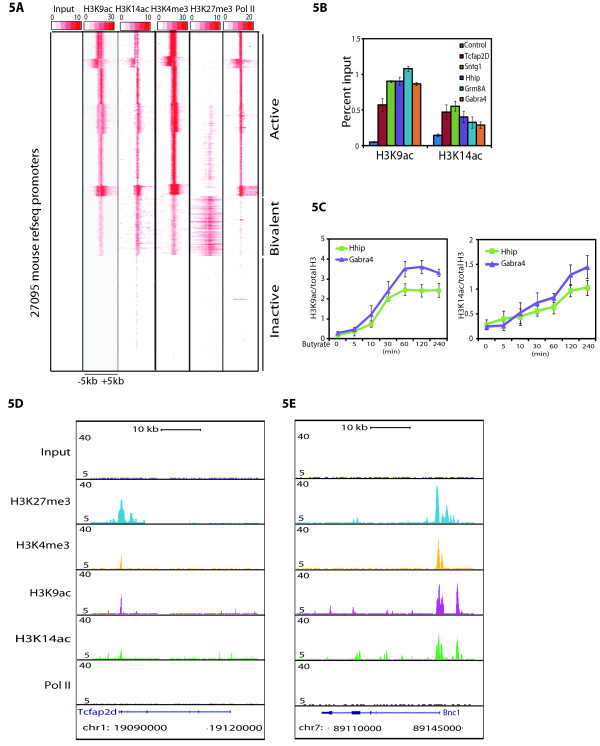
**H3K9ac and H3K14ac associate with active as well as bivalent promoters. **(**A**) Heatmap of the signal density using k-means clustering observed on 27095 mouse refseq promoters (-/+5 kb) for H3K9ac and H3K14ac along with H3K4me3 and H3K27me3 (hallmark of bivalent promoters) and Pol II. The clustering of density map shows three different categories of genes. Active promoters having H3K9ac, H3K14ac, H3K4me3 and Pol II, bivalent promoters showing H3K4me3 and H3K27me3 along with H3K9ac and H3K14ac, and inactive promoters lacking all above marks along with Pol II. (**B**) Presence of H3K9ac and H3K14ac over randomly chosen bivalent loci was validated by ChIP-qPCR. (**C**) Increase in the H3K9 and H3K14 acetylation over bivalent promoters (Hhip and Gabra4) following HDAC inhibition by sodium butyrate. The presence of H3K9ac and H3K14ac over these bivalent loci at the indicated time points after the sodium butyrate treatment was measured by ChIP-qPCR. ChIP signals for H3K9ac and H3K14ac were normalized to total H3. Primer sequences used in ChIP-qPCR is provided in Additional file 4: Table S1. Error bars represent the standard deviation for three technical replicates. (**D** and **E**) UCSC genome browser track of two representative examples of loci showing H3K9ac and H3K14ac over the bivalent promoters.

In order to test the presence of H3K9 and H3K14 acetylations over the bivalent promoters, all the 27095 mouse promoters from UCSC genome browser were taken [28] and subjected to k-means clustering using seqMiner [33]. H3K4me3 and H3K27me3 (dual hallmarks of bivalent promoters) were used to get the bivalent loci. Pol II was included in the clustering to differentiate active and inactive genes. The resulting heatmap is shown in Figure 5A. In agreement with previous genome-wide studies, there are three distinct categories of loci. In respect to the studied acetylation (i) active loci marked by Pol II, H3K4me3, and devoid of strong H3K27me3 signals are highly enriched in H3K9ac and H3K14ac, (ii) bivalent loci, which are marked by H3K4me3, H3K27me3 and low Pol II signals contain both H3K9ac and H3K14ac signals, and (iii) inactive loci, on which either of the above signals is missing. Our analysis suggests that indeed on bivalent promoters H3K9 and H3K14 acetylations occur together with H3K4 and H3K27 trimethylations (Figure 5A). 

To test the presence of H3K9ac and H3K14ac over bivalent promoters, we took a subset of randomly selected loci and successfully validated them by ChIP-qPCR (Figure 5B). Further to test if H3K9ac and H3K14ac are functional at these bivalent loci, a HDAC inhibitor and followed the level of H3K9ac and H3K14ac over time. First, Oct4 levels were monitored by Western blot to assess the pluripotent state of the ES cells at various time points after sodium butyrate treatment. We found that Oct4 levels are comparable in non-treated and sodium butyrate treated ES cells at various time points ( Additional file 7: Figure S6), suggesting that the pluripotent state of the cells did not change during the treatment. Next we analyzed two selected loci for the presence of H3K9ac and H3K14ac during the sodium butyrate treatment. We found that inhibition of HDACs by sodium butyrate leads to increase in H3K9ac and H3K14ac suggesting that these marks are indeed functionally deposited and are actively maintained (Figure 5C). UCSC genome browser tracks of two representative examples on previously characterized bivalent promoters [8] harboring the H3K9ac and H3K14ac over the promoters along with H3K4me3 and H3K27me3 are shown in Figure 5D and 5E. Thus, our genome-wide analyses show that H3K9ac and H3K14ac mark bivalent promoters along with active (H3K4me3) and repressive (H3K27me3) histone marks over developmentally regulated genes in undifferentiated ES cells.

### Differential correlation of H3K14ac with repressive histone marks as compared to H3K9ac

**Figure 6:**
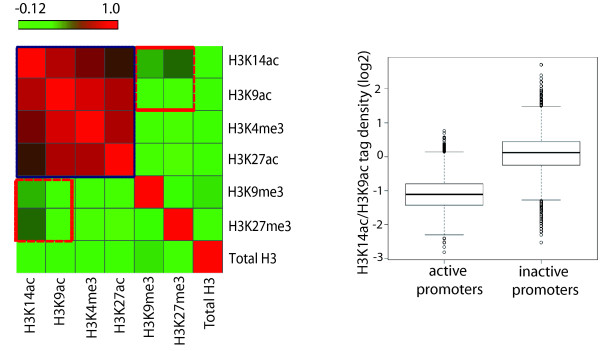
**Differential H3K14ac over inactive promoters as compared to H3K9ac. **(**A**) Heatmap representing the correlation between total H3, H3K9ac, H3K14ac, H3K4me3, H3K27ac, H3K27me3 and H3K9me3 around the TSS (-/+ 2 kb). Active promoter marks; H3K14ac, H3K9ac, H3K4me3 and H3K27ac clustered separately (blue square) to form an active promoter chromatin state. Inactive marks (H3K9me3 and H3K27me3) have higher co-occurrence with H3K14ac as compared to H3K9ac (red square) (**B**) Ratio of H3K14ac/H3K9ac ChIP-seq tag density plotted for active promoters and inactive promoters. Ratio is significantly higher for inactive promoters suggesting that level of H3K14ac is higher over inactive promoters as compared to H3K9ac.

We next analyzed co-localization of the H3K9 and H3K14 acetylation marks with various active and repressive histone modifications. The Pearson correlation coefficient was calculated for these modifications in a window of 2 kb upstream and downstream the TSSs of all the mouse refseq genes (27095). Heatmap of various histone modifications suggest that active histone marks have high correlation and are grouped together for efficient gene expression (Figure 6A, blue square). Surprisingly, we observed higher correlation of H3K14ac with various repressive marks, such as H3K27me3 and H3K9me3, when compared to H3K9ac (Figure 6A, red square). To further test the association of H3K14ac with inactive marks, H3K27me3 and H3K9me3, we took 7924 inactive genes, which lack Pol II and H3K4me3 (Figure 5A) and calculated the ratio of H3K14ac/H3K9ac tag density. The ratio of H3K14ac/H3K9ac is significantly higher over inactive genes as compared to active genes suggesting that H3K14ac is specifically and significantly enriched at inactive genes (Figure 6B). In Figure 6B we used H3K4me3, Pol II signals distinguish active TSSs from inactive ones. To further strengthen our observations we took the 500 weakest (or not expressed) and the 500 highest expressed gene promoters, but this time based on their expression profiles as calculated from RNA-seq data (kindly provided by the D. Schübeler), and calculated the ratio of H3K9 and H3K14 acetylation tag densities over the least expressed and highly expressed promoters. This analysis again shows that H3K14ac is specifically enriched at the weakest (or inactive) promoters as compared to H3K9ac ( Additional file 8: Figure S7). This in turn suggests that H3K14ac, which is generally considered as a mark of active promoters along with other acetylation marks, can also mark inactive promoters, although to a lesser extent.

### A subset of inactive genes is specifically enriched for H3K14ac

**Figure 7:**
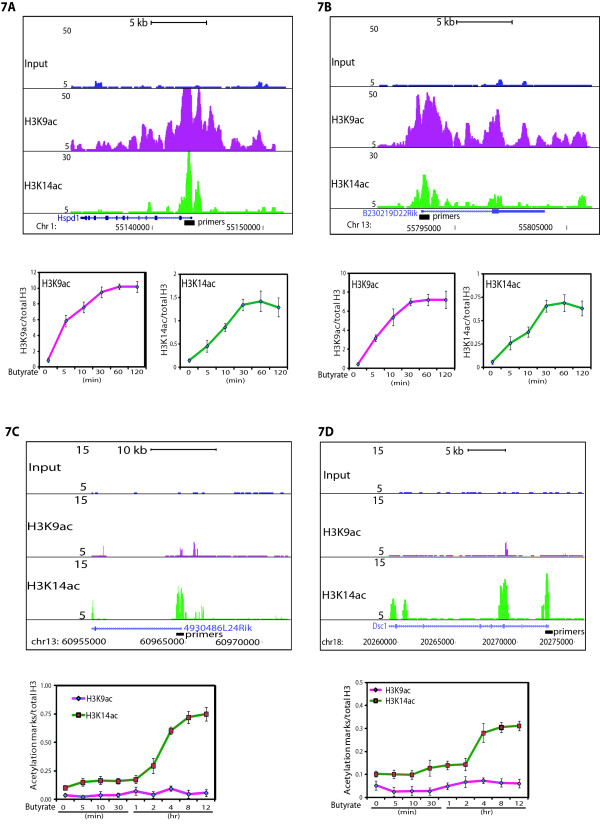
**Selective increase in H3K14ac over inactive genes in presence of sodium butyrate. **(**A**, **B**) Rapid increase in the H3K9 and H3K14 acetylation over active promoters (Hspd1 and B230219D22Rik) caused by HDAC inhibitor. The upper panels show the UCSC genome browser tracks for the genes analysed. (**C**, **D**) Selective increase in H3K14ac over the inactive promoters (4930486L24Rik and Dsc1) as compared to H3K9ac following the treatment with HDAC inhibitor. The upper panels show the UCSC genome browser track for the genes analysed. The presence of H3K9ac and H3K14ac over these loci at the indicated time points after the sodium butyrate treatment was measured by ChIP-qPCR. ChIP signals for H3K9ac and H3K14ac were normalized to total H3. Positions of the primers used for ChIP-qPCR is shown in the UCSC genome browser track and primer sequences are provided in Additional file 4: Table S1. Error bars represent the standard deviation for three technical replicates.

**Figure 8:**
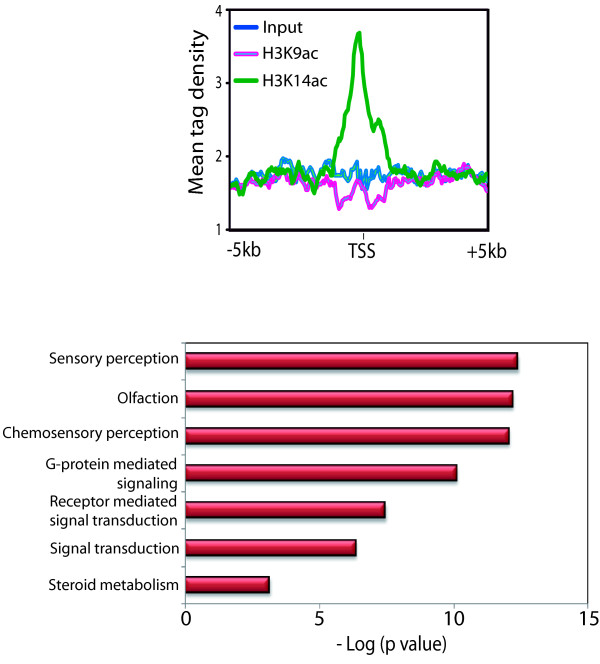
**A subset of inactive promoters having high H3K14ac is poised for stimuli dependent activation. **(**A**) Average ChIP-seq profile of 500 inactive genes around the transcription starts sites (-/+ 5 kb) shows specific enrichment of H3K14ac as opposed to H3K9ac. (**B**) Gene ontology (using David Bioinformatics, http://david.abcc.ncifcrf.gov/) analysis of 500 inactive genes having high H3K14ac suggest that these genes are activated in a stimuli dependent manner. The gene ontology term is on the y axis, and the negative log of p value indicating significance of enrichment is on the x axis.

To understand the functionality of H3K14ac over inactive promoters, we examined the level of this histone mark over active and inactive genes in presence of sodium butyrate, a HDAC inhibitor, and compared it to H3K9ac. In agreement with our above observations, we found that active genes exhibited a remarkable increase in H3K9ac and H3K14ac in presence of sodium butyrate (Figure 7A and 7B). However, examination of H3K9ac and H3K14ac level at inactive genes in presence of sodium butyrate revealed a slow and selective increase in H3K14ac, while at these sites H3K9ac did not change (Figure 7C and 7D). The selective increase in H3K14ac at inactive genes in presence of sodium butyrate suggests that inactive genes are subjected to constant H3K14 acetylation and deacetylation. This dynamic H3K14ac at inactive genes may poise them for future activation. To test this hypothesis, we selected 500 inactive genes having higher H3K14ac as compared to H3K9ac (Figure 8A) and subjected them to gene ontology (Figure 8B). Our analysis suggests that inactive genes, which are having significant H3K14ac over H3K9ac belong to various pathways that are induced by various stimuli such as sensory perception, olfaction and chemosensory perception. Other pathways include receptor activities, which are induced upon ligand binding to activate the signal transduction and ultimately cellular responses (Figure 8B). Thus, our data indicate that H3K14ac is selectively present over a subset of non-expressed genes and HDACs frequently remove this mark to keep the genes inactive. The coordinated action of HATs and HDACs may poise these genes for stimuli dependent activation.

## Discussion

### H3K9ac and H3K14ac co-occur with other “active” histone modifications establishing a chromatin conformation that is compatible with transcription

Recent genome-wide studies have generated comprehensive chromatin landscapes for various transcription factors and histone modifications [3,14,35]. In our study, by using a new specific H3K14ac antibody, we show that H3K9ac and H3K14ac co-occur with other active histone modifications, H3K4me3 and H3K27ac in mES cells. Thus, it seems that at these sites all these “active” histone modifications are deposited by the corresponding activities sequentially, or at the same time, to act in a coordinated way. A possible mechanistic cross-talk between H3K4me3 and H3 acetylation could occur in the following steps: (i) histone lysine methyltransferases (HMT), Set1/COMPASS associates with the early initiating RNA Pol II through the PAF1 complex to methylated histone H3K4 around promoters [36]; (ii) various HAT complexes (such as p300/CBP, NuA3, NuA4) would then recognize this H3K4me3 indirectly or directly [37] and acetylate the chromatin; (iii) in parallel or subsequently other HATs, such as GCN5/PCAF-containing SAGA and ATAC complexes, may recognize the H3K4me3 by the Tudor domain of their SGF29 subunit [38,39]. This cross-talk between the H3K4me3 and H3 acetylation is supported by the observations indicating that knockdown of WDR5 (subunit of several chromatin modifying complexes including HMTs and HATs) or Set1 (catalytic subunit) of H3K4 methyltransferases complex not only decrease the H3K4me2/3, but they also decrease the acetylation levels at given promoters [40-42]. Thus, H3K4me3 seems to provide a binding platform for HATs, which are specific for the H3 tails, but may not be specific for the given lysine residue. This in turn would explain the fact that we see the acetylation of various H3 residues together (such as H3K9, H3K14 and H3K27) at the 5’ end of the genes, which follow the presence of H3K4me3 and the underlying CpG islands (see below). This would suggest that some marks (i.e. H3K9ac and H3K14ac) are establishing more the openness of the chromatin, while others (i.e. H3K4me3) may serve more as a docking site [43,44]. Thus, all these marks together may be required to regulate Pol II transcription initiation positively.

### The presence of H3K9ac and H3K14ac at promoters correlates with their CpG content

Approximately 70% of all the annotated gene promoters are associated with a CpG islands [27], which have distinct patterns of chromatin configuration. Interestingly, in mES cells CXXC finger protein 1 (Cfp1), a component of Setd1 histone methyltransferases complex has a preference for CpG clusters and can be recruited to artificial promoter-less CpG clusters that subsequently lead to trimethylation of H3K4, a CpG island promoter signature [45,46]. The presence of H3K4me3 might be followed by a cascade of events and consequent acetylation of different H3 and/or H4 residues (as discussed above). Acetylation (H3K9 and H3K14) of the nucleosomes associated with the CpG islands might be acting as a physical barrier to inhibit the H3K9me3 of the chromatin [47]. Housekeeping gene promoters have generally high CpG content and are often protected from DNA methylation and methylation of H3K9, enabling constitutive expression of the associated genes. Thus, our findings which show a very good correlation between CpG content of promoter regions and acetylation of the associated nucleosomes on H3K9 and H3K14, along with H3K4me3 are in a good agreement with the model in which all these “active” histone marks influence the local chromatin structure to simplify the regulation of gene activity (reviewed in [27]). Our results also suggest the dependence of histone modifications such as H3K4me3, H3K9ac and H3K14ac on the DNA sequences underneath.

### H3K9 and H3K14 acetylation label active enhancers together with H3K27 acetylation

Histone modification profiles have been used for identifying enhancer elements [4]. Enhancers are characterized by high H3K4me1/H3K4me3 ratio, open chromatin, low Pol II and presence of HAT co-activators such as p300 [9] and ATAC [10]. Interestingly, chromatin modification patterns at enhancers are much more variable and cell type specific, than chromatin patterns at promoters that are much more conserved [9,10]. Our study establishes that H3K9ac and H3K14ac also mark enhancers. Furthermore, like H3K27ac [11], these two marks can differentiate between the active and inactive/poised enhancers (Figure 4). RNA Pol II from these active enhancers produces bidirectional short (<2 kb) non-coding eRNAs, whose expression level correlate with the proximal gene activity. [30]. Thus, along with H3K27ac, H3K9ac and H3K14ac can be used to discriminate the active enhancers from the inactive/poised enhancers at global and gene specific level.

### Genome-wide bivalent promoters are also marked by H3K9 and H3K14 acetylation

Here we also show that bivalent promoters are not only marked by the H3K4me3 and H3K27me3, but also by the other active histone marks, such as H3K9ac and H3K14ac. There are two possibilities for the presence of H3K9ac and H3K14ac over the bivalent promoters. First, both acetylation marks may be required in addition to H3K4me3 for preparing these promoters of developmental genes for rapid induction at later stages. Second, the presence of H3K9ac and H3K14ac over bivalent promoters might act as a physical barrier and thereby inhibiting the trimethylation of H3K9, which eventually can lead to a stable silencing of developmentally regulated genes [48]. Thus acetylation of H3K9 and H3K14 may be required as a balancing mechanism to inhibit the permanent transcriptional silencing of the developmentally regulated genes [47]. Our genome-wide study showing presence of H3K9ac at bivalent promoters is in accordance with earlier studies describing the presence of H3K9ac, along with the active (H3K4me3) and repressive (H3K27me3) chromatin marks, over bivalent promoters in undifferentiated ES cells [49]. Note however that Hezroni et al. [50] observed only very low H3K9ac levels over bivalent gene promoters in ES cells. The difference observed between H3K9ac enrichment over bivalent promoters in our data and that of Hezroni et al. [50] could be attributed to the fact that our ChIP-seq data shows an increased depth in the coverage of H3K9ac sites compared to the one obtained previously. Indeed, we obtained 23 million reads for our H3K9ac ChIP seq data versus 14 million reads in the former study. Higher sequencing depth will result in a more complete coverage of the identified H3K9ac signals and also identify smaller peaks. Thus, it is possible that we detect more peaks that would allow us a more thorough analysis. Alternatively, differential enrichment of H3K9ac over bivalent promoters in our study and that of Hezroni et al. [50] is also possible because of the different antibodies used in the two studies, which can have distinct specificities.

### A subset of inactive promoters are specifically enriched for H3K14ac

H3K9ac and H3K14ac are associated with active promoters and are considered as hallmarks of active transcription. However, we observed that in contrast to H3K9ac, H3K14ac is selectively and specifically associated with repressive marks such as H3K9me3 and H3K27me3 on a subset of inactive promoters. Presence of H3K14ac on inactive promoters along with H3K9me3 and H3K27me3 is in agreement with previous observation where high throughput characterization of combinatorial histone marks using proteomic approach shows highest abundance for H3K14ac peptide with activation and repressive marks [51]. Our data suggest that HATs may bind transiently to a subset of inactive promoters and acetylated H3K14, which is then removed by the transient binding of HDACs. Inhibition of HDACs leads to substantial increase in H3K14ac of these inactive promoters, suggesting that these promoters are subjected to dynamic regulation by HATs and HDACs. Transient acetylation and deacetylation of these promoters may keep these promoters unexpressed but at the same time primed for future activation upon receiving the external stimuli. It is thus possible that the presence of H3K14ac over a subset of inactive promoters may serve as a landing platform for further HATs, which have bromodomains, such as GCN5/PCAF, and propagate the acetylation of the given loci at a later stage. Thus, presence of H3K14ac and the consequent coordinated action of HATs and HDACs over these inactive genes might poise them for stimuli dependent future activation.

## Conclusions

In this study, we characterized the H3K9 and H3K14 acetylations over various genomic regions in mES cells. Taken together our data suggest that genome-wide coordinated modifications of histone acetylation (i.e. H3K9, H3K14 and H3K27) and methylation (H3K4me3) provide a general signature for facilitating gene expression over active promoters. On the other hand we showed differential enrichment of H3K14ac over inactive promoters. Furthermore our analyses suggest correlation between acetylation of nucleosomes over the promoters and the underlying sequences and demonstrate the presence of H3K9ac and H3K14ac over enhancers, which can be used in the future studies to discriminate active enhancers from inactive/poised ones. In conclusion, our comprehensive study of H3K9ac and H3K14ac demonstrates a wider role for these to marks in gene regulation than originally thought.

## Methods

### Cell culture

Wild type embryonic stem cells were derived from blastocysts (3.5 PC) and cultivated on feeder cells (37°C, 5% CO_2_) in DMEM (4.5 gm/lit), 15% FCS, leukemia inhibiting factor, penicillin/streptomycin, L-glutamine, and non-essential amino acids. At least three passages under feeder free conditions on 0.1% gelatin were used to exclude feeders.

### Antibodies

Mouse monoclonal anti-H3K14ac antibody (13HH3-1A5) was produced against the peptide “STGGK(ac)APRKC”. The characterization of the antibody is shown in Additional file 2: Figure S2. The H3K9ac antibody is from Abcam -ab4441.

### Sodium butyrate treatment of mES cells

mES cells were treated with 5 mg/ml sodium butyrate for 0 min, 5 min, 10 min, 30 min, 1 hr, 2 hr, 4 hr, 8 hr and 12 hr. Cells were cross linked with 1% formaldehyde and ChIP was performed as described below. Level of Oct4 was monitored by Western blot to see the pluripotent state of the ES cells at various time points after sodium butyrate treatment ( Additional file 7: Figure S6). Similar level of Oct4 at all time points suggest that the time points we have used for sodium butyrate treatment have no effect on pluripotency of the ES cells. Tubulin was used as a loading control. Anti-Oct4 antibody (611202) used in the Western blot is from BD labs.

### Chromatin Immunoprecipitation (ChIP) and sequential ChIP

All ChIP experiments were carried out on 2X10^7^ cells per antibody. Cells were cross-linked with 1% formaldehyde, lyses and syndicated in sonication buffer (10 mM Tris–HCl pH 7.5, 200 mM NaCl, 1% SDS, 4% NP-40, 1 mM PMSF) to obtain an average chromatin size of 200-500 bp. Chromatin was pre-cleared using 50 μl of a 50% protein A sepharose (GE healthcare) slurry for 1 h at 4°C with gentle inverting. Immunoprecipitation were carried out in 10 ml of IP buffer (20 mM Tris–HCl pH 8.0, 150 mM NaCl, 2 mM EDTA, 1% Triton-X 100). 15 μl of ZZZ3 and SPT20 serum antibodies were used. Input chromatin was obtained after pre-clearing, by de-cross linking and purifying input DNA using a Qiaquick column (Qiagen) according to manufacturer’s instructions. Immunoprecipitation were carried out with inverting at 4°C for 14–16 h. The samples were then incubated with 50 μL of a 50% Protein A sepharose slurry for 3 h at 4°C with gentle inverting. IP samples were reverse-cross linked and the DNA was purified using a Qiaquick column (Qiagen). Q-PCR using SYBR green was used to validate known target sites before and after sequencing. For sequential ChIP, at least four ChIP assays of 2X10^7^ cells were used for the first IP (H3K9ac). Following standard washing, elution was performed with 10 mM DTT (30 min, 37°C). The eluates from four ChIPs were combined, diluted at least 30 times with ChIP dilution buffer and secondary antibody (H3K14ac) was incubated overnight. The subsequent steps were performed as for regular ChIPs. Validation of ChIP-seq was performed by the assessment of the individual enrichment over the control genomic region by ChIP-qPCR in triplicate with primers specific for these regions using SYBR Green master mix (Roche).

### Chromatin Immunoprecipitation – High throughput-sequencing (ChIP-seq)

#### Library preparation and sequencing

We followed the manufacturer's (Solexa) protocol for creating genomic DNA libraries as previously described in [52]. ChIP DNA and input DNA were first band-isolated on a 2% agarose to obtain fragments between 150 and 350 base pairs and DNA was extracted using the QIAquick gel extraction kit (Qiagen) and eluted in 34 μl. After end-repair and addition of a single adenosine ("A") nucleotide, adapters were ligated to samples for 15 min at room temperature in the following fashion: the samples eluted from the MinElute column in 10 μL were ligated to 1 μl of adapters using 1.3 μL of LigaFast T4 DNA Ligase (3 Units/μl; Promega) and 12.3 μL Rapid Ligation Buffer (Promega). For DNA libraries, the Illumina genomic DNA adapters were diluted 1:10. After 15 min, samples were purified with the MinElute PCR purification kit (Qiagen).

Adapters in excess were eliminated by using gel purification on a 2% agarose E-Gel (Invitrogen) for 20 min, together with Track-It 50 bp DNA ladder (Invitrogen). DNA fragments ranging from 150 base pairs to 500 base pairs were extracted and recovered in 28 μl EB with a QIAquick gel extraction kit (Qiagen). To amplify the library, PCR was performed using Illumina genomic DNA primer "1.1" and Illumina genomic DNA primer "2.1" with 15 cycles (Input DNA) or 17 cycles (ChIP DNA) of amplification. A final size selection was performed using a 2% agarose E-Gel to obtain a library with a median length of ~230 bp which is within the recommended size range for cluster generation on Illumina's flow cell. The library was recovered in 20 μl EB using MinElute Gel Extraction kit (Qiagen). Finally, DNA concentrations and purities (A260/280 nm ratios) were measured on a Nanodrop spectrophotometer. Sequencing was carried out on the Illumina (Solexa) platform at a sequencing depth of 1 lane averaging 10 million reads, read length of 27 + bp, single-end reads and mapped to mouse genome build (mm9).

#### Establishment of list of reference loci

ChIP-seq data were mapped using the ELAND software (Illumina) allowing one mismatch. Mapped read data were used as an input and to establish list of loci using MACS (Using default parameters except: mfold 12; tag size according to platform; band width 100) [23]. Input DNA file was used as a control in all peak detection analyses. To calculate a single enrichment value for a binding site, tag density is defined as the number of tags present or overlapping in user-defined window around the reference site. The algorithm for the signal enrichment calculation is described in details in [33].

#### Average profile calculations

We extracted the tag density in a 5 kb window surrounding the TSSs and gene body using the program seqMINER which generates heatmap as well as the profiles [33]. The sequenced ChIP-seq reads represent only the end of each immunoprecipitated fragments instead of the precise protein-DNA binding sites. To illustrate the entire DNA fragment, basically before analysis, 3^′^ end of each ChIP-seq read was extended to 200 bp in the direction of the reads. For average gene profiles, genes (+/-5000 bp from binding site) were divided in 100 bins of length relative to the gene length. Moreover 10 equally sized (50 bp) bins were created on the 5’ and 3’ of the gene and ChIP-seq densities were collected for each dataset in each bin. The average of enrichments over input in each bin was plotted for each dataset. For average TSS profile, the genes as above were aligned on their TSSs and densities were collected in 100 equally sized bins around the TSS (-/+5000 bp).

#### Identification of intergenic regions

The list of refseq genes was obtained from UCSC table browser [28]. Co-ordinates for the intergenic regions were extracted from refseq genes using complement intervals of a dataset tools from Galaxy web server ( https://main.g2.bx.psu.edu/). All those regions, which do not correspond to the beforehand defined promoter regions, that are not gene bodies, and are not regions that are situated 2 kb downstream of the end of annotated genes, are considered as intergenic regions. Overlapping piece of intervals (from Galaxy web tools) with an overlap of at least 500 bps was used to distinguish peaks, which are intergenic for H3K9 and H3K14 acetylation.

#### Data source

ChIP-seq datasets were downloaded from the public data bank Gene Expression Omnibus ( http://www.ncbi.nlm.nih.gov/gds) under the accession number: GSM307618 (H3K4me3-mES); GSM307619 (H3K27me3-mES); GSM307621 (H3K9me3-mES); GSM594577 (H3K4me1-mES); GSM594578 (H3K27ac-mES); GSM307623 (Pol II-mES); GSM307624 (total H3-mES); GSM307625 (input-mES); GSM699164 (p300-mES). The sequencing data we have generated is deposited in the GEO database under the accession number GSM775313 (H3K9ac-mES) and GSM775314 (H3K14ac-13HH3-1A5-mES). CpG island definition and locations of known genes were downloaded from UCSC genome browser [28]. Number of mapped reads for each data set is provided in the Additional file 9: Table S2.

Link to allow review of record:

http://www.ncbi.nlm.nih.gov/geo/query/acc.cgi?token=nhclxooaewkmqvq&acc=GSE31284.

### Creation of density files for genome browser data visualization

Raw BED files are used as input for ad hoc (WIG) density file creation script as described in [33]. Reads are directionally extended of their theoretical length (200 bp), and 25 bp bins are created. In each bin, the maximal number of overlapping reads is computed. Tracks were uploaded and displayed using fixed scale representation in the UCSC genome browser [28].

### Data analysis

The scatter plots, k-means clustering and average gene profiles are created using seqMiner [33]. Box plots and correlation analysis are produced using R software ( http://r-project.org/). The distribution of H3K9ac and H3K14ac in various genomic regions is calculated using the CEAS software [22]. Briefly, CEAS estimates the relative enrichment level of ChIP regions in each gene feature with respect to the whole genome. In our analysis promoters correspond to the 2 kb upstream regions from the transcription start site (TSS) of refseq genes. All those regions, which do not correspond to the beforehand defined promoter regions, that are not gene bodies, and are not regions that are situated 2 kb downstream of the end of annotated genes, are considered as intergenic regions (more details are available at http://liulab.dfci.harvard.edu/CEAS/usermanual.html and [22]). Gene ontology was performed using David Bioinformatics tools ( http://david.abcc.ncifcrf.gov/home.jsp).

## Abbreviations

mES: Mouse embryonic stem cells; HAT: Histone acetyl transferase; HDAC: Histone deacetylases; ChIP: Chromatin immunoprecipitation; ChIP-seq: Chromatin immunoprecipitation coupled high throughput sequencing; mAbs: Monoclonal antibodies; TSSs: Transcription start sites; CpG: Cytosine-phosphate diester-guanine; PHD: Plant homeodomain; eRNA: Enhancer RNA; Pol II: RNA Polymerase II.

## Competing interests

The authors declare that they have no competing interests.

## Authors’ contributions

KK generated ChIP-seq data and performed experiments. KK, ARK and LT analyzed data. MOA and HK generated antibodies. KK and LT wrote the manuscript. LT planned, coordinated and supervised the project. All authors read and approved the manuscript.

## Supplementary Material

Additional file 1 **Figure S1. **The commercially available anti-H3K14ac antibody (Upstate (07-353)) cross-reacts with other peptides. Enzyme linked immunosorbent assay (ELISA) using various peptides such as H3K14 acetylated (H3K14ac) and non-acetylated (H3K14) and non specific histone H4 acetylated peptide (H4K5ac-K12ac) suggest that anti-H3K14ac antibody from Upstate (07-353) cross-reacts with non-acetylated H3K14 peptide as well as with H4K5andK12ac peptide. Click here for file

Additional file 2 **Figure S2. **Characterization of mouse monoclonal anti-H3K14ac (13HH3-1A5) antibody. (A) Western blot analysis using the 13HH3-1A5 (anti H3K14ac antibody) on the recombinant E. coli expressed histone H3 and histones extracted by and acidic extraction protocol from human HeLa cells (right panel). Coomassie blue stained SDS-PAGE of the proteins used for the western blot analysis in a 100-fold dilution. (B) Enzyme linked immunosorbent assay (ELISA) using various peptides such as H3K14 acetylated (H3K14ac) and non-acetylated (H3K14), H3K14 dimethylated, H3S10 phosporylated and K14 dimethylated (H3pS10K14dimethyl), H3S10 phosporylated (H3pS10), H3K9 dimethylated (H3K9dimethyl), H3S10 phosporylated and K9 dimethylated (H3pS10K9dimethyl) and histone H4 acetylated peptide (H4K5, 8, 12 and 16 ac). Click here for file

Additional file 3 **Figure S3. **Validation of (A) H3K9ac and (B) H3K14ac ChIP-seq in mouse embryonic stem cells by ChIP-qPCR. Peaks of local enrichment of H3K9 and H3K14 acetylation were determined after sequence alignment and normalization to input DNA. Tag density per peak (shown on the right of the graph) is plotted as fold enrichment over an arbitrarily chosen control genomic region. The list of primer is provided as Supplementary Table S1. These ChIP-qPCR results confirmed the specificity of the predicted peaks for H3K9 and H3K14 acetylation. Click here for file

Additional file 4 **Table S1. **List of primers used for ChIP-qPCR validation. Click here for file

Additional file 5 **Figure S4. **Enrichment of H3K9ac and H3K14ac over various genomic regions. Average enrichment profile of H3K9 and H3K14ac over the promoters, coding exons, introns and distal intergenic regions. Enrichment over distal intergenic and intronic sites is comparable to promoters. Click here for file

Additional file 6 **Figure S5. **Correlation of H3K9ac (A) and H3K14ac (B) intergenic peaks with H3K4me1, H3K27ac, Pol II and p300. Heatmaps of the signal density using k-means clustering over H3K9ac and H3K14ac distal intergenic sites (-/+ 5 kb). Strong correlation with H3K4me1 and H3K27ac and presence of Pol II and p300 over H3K9ac and H3K14ac intergenic sites suggest that they may act as enhancers. Click here for file

Additional file 7 **Figure S6. **Measurement of Oct4 level at various time points after the treatment with 5 mg/ml sodium butyrate in ES cells. Level of Oct4 was monitored by Western blot to see the pluripotent state of the ES cells at various time points after sodium butyrate treatment. The similar levels of Oct4 observed at different experimental time points suggest that the time points we have used for sodium butyrate treatment have no effect on pluripotency of the ES cells. Tubulin was used as a loading control. Click here for file

Additional file 8 **Figure S7. **Ratio of tag density of least expressed to highest expressed gene promoters for H3K9 and H3K14 acetylation. H3K9 and H3K14 acetylation tag density were calculated over 500 least and highest expressed gene promoters based upon their RNA-sequencing expression profile. The ratio of tag density of least expressed to highest expressed plotted for H3K9 and H3K14 acetylation, which shows that H3K14ac is specifically enriched at inactive promoters as compared to H3K9ac. Click here for file

Additional file 9 **Table S2.**Number of mapped reads for the data set used in this study. Click here for file
