# Supplementary material for: H3K9 and H3K14 acetylation co-occur at many gene regulatory elements, while H3K14ac marks a subset of inactive inducible promoters in mouse embryonic stem cells
Source: BMC Genomics. 2012 Aug 24;13:424. doi: 10.1186/1471-2164-13-424 (PMC3473242; doi:10.1186/1471-2164-13-424)
Supplement: Additional file 1 — Figure S1. The commercially available anti-H3K14ac antibody (Upstate (07-353)) cross-reacts with other peptides. Enzyme linked immunosorbent assay (ELISA) using various peptides such as H3K14 acetylated (H3K14ac) and non-acetylated (H3K14) and non specific histone H4 acetylated peptide (H4K5ac-K12ac) suggest that anti-H3K14ac antibody from Upstate (07-353) cross-reacts with non-acetylated H3K14 peptide as well as with H4K5andK12ac peptide. [file 1471-2164-13-424-S1.doc]

**Additional Files**

**Additional File 1: Supplementary Figure S1. The commercially available anti-H3K14ac antibody (Upstate (07-353)) cross-reacts with other peptides.** Enzyme linked immunosorbent assay (ELISA) using various peptides such as H3K14 acetylated (H3K14ac) and non-acetylated (H3K14) and non specific histone H4 acetylated peptide (H4K5ac-K12ac) suggest that anti-H3K14ac antibody from Upstate (07-353) cross-reacts with non-acetylated H3K14 peptide as well as with H4K5andK12ac peptide.

**
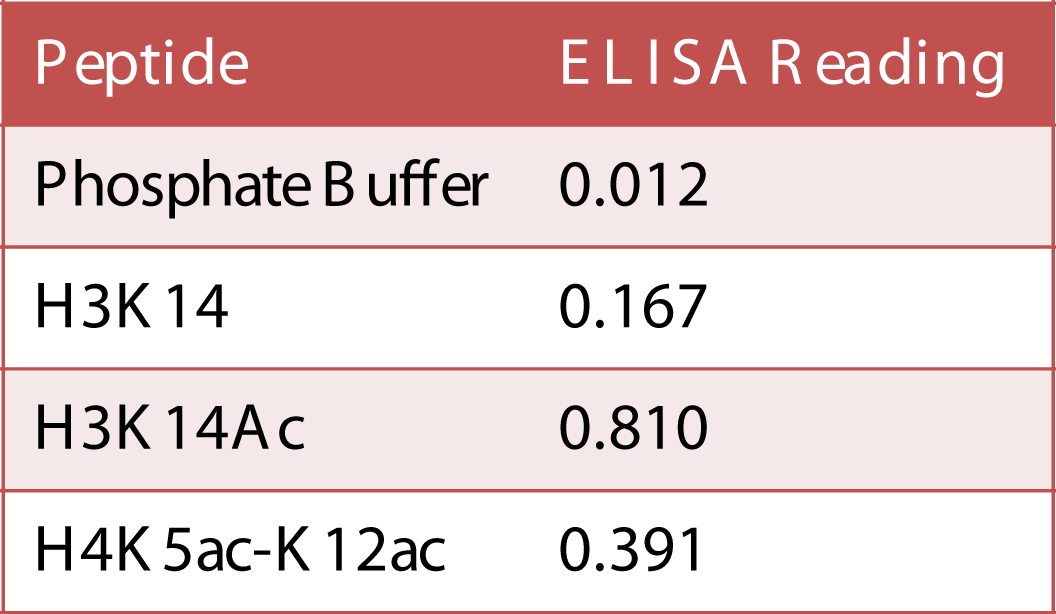
**
