# Supplementary material for: H3K9 and H3K14 acetylation co-occur at many gene regulatory elements, while H3K14ac marks a subset of inactive inducible promoters in mouse embryonic stem cells
Source: BMC Genomics. 2012 Aug 24;13:424. doi: 10.1186/1471-2164-13-424 (PMC3473242; doi:10.1186/1471-2164-13-424)
Supplement: Additional file 2 — Figure S2. Characterization of mouse monoclonal anti-H3K14ac (13HH3-1A5) antibody. (A) Western blot analysis using the 13HH3-1A5 (anti H3K14ac antibody) on the recombinant E. coli expressed histone H3 and histones extracted by and acidic extraction protocol from human HeLa cells (right panel). Coomassie blue stained SDS-PAGE of the proteins used for the western blot analysis in a 100-fold dilution. (B) Enzyme linked immunosorbent assay (ELISA) using various peptides such as H3K14 acetylated (H3K14ac) and non-acetylated (H3K14), H3K14 dimethylated, H3S10 phosporylated and K14 dimethylated (H3pS10K14dimethyl), H3S10 phosporylated (H3pS10), H3K9 dimethylated (H3K9dimethyl), H3S10 phosporylated and K9 dimethylated (H3pS10K9dimethyl) and histone H4 acetylated peptide (H4K5, 8, 12 and 16 ac). [file 1471-2164-13-424-S2.doc]

**Additional File 2: Supplementary Figure S2. Characterization of mouse monoclonal anti-H3K14ac (13HH3-1A5) antibody. (A)** Western blot analysis using the 13HH3-1A5 (anti H3K14ac antibody) on the recombinant E. coli expressed histone H3 and histones extracted by and acidic extraction protocol from human HeLa cells (right panel). Coomassie blue stained SDS-PAGE of the proteins used for the western blot analysis in a 100-fold dilution. (B) Enzyme linked immunosorbent assay (ELISA) using various peptides such as H3K14 acetylated (H3K14ac) and non-acetylated (H3K14), H3K14 dimethylated, H3S10 phosporylated and K14 dimethylated (H3pS10K14dimethyl), H3S10 phosporylated (H3pS10), H3K9 dimethylated (H3K9dimethyl), H3S10 phosporylated and K9 dimethylated (H3pS10K9dimethyl) and histone H4 acetylated peptide (H4K5, 8, 12and 16ac).

**
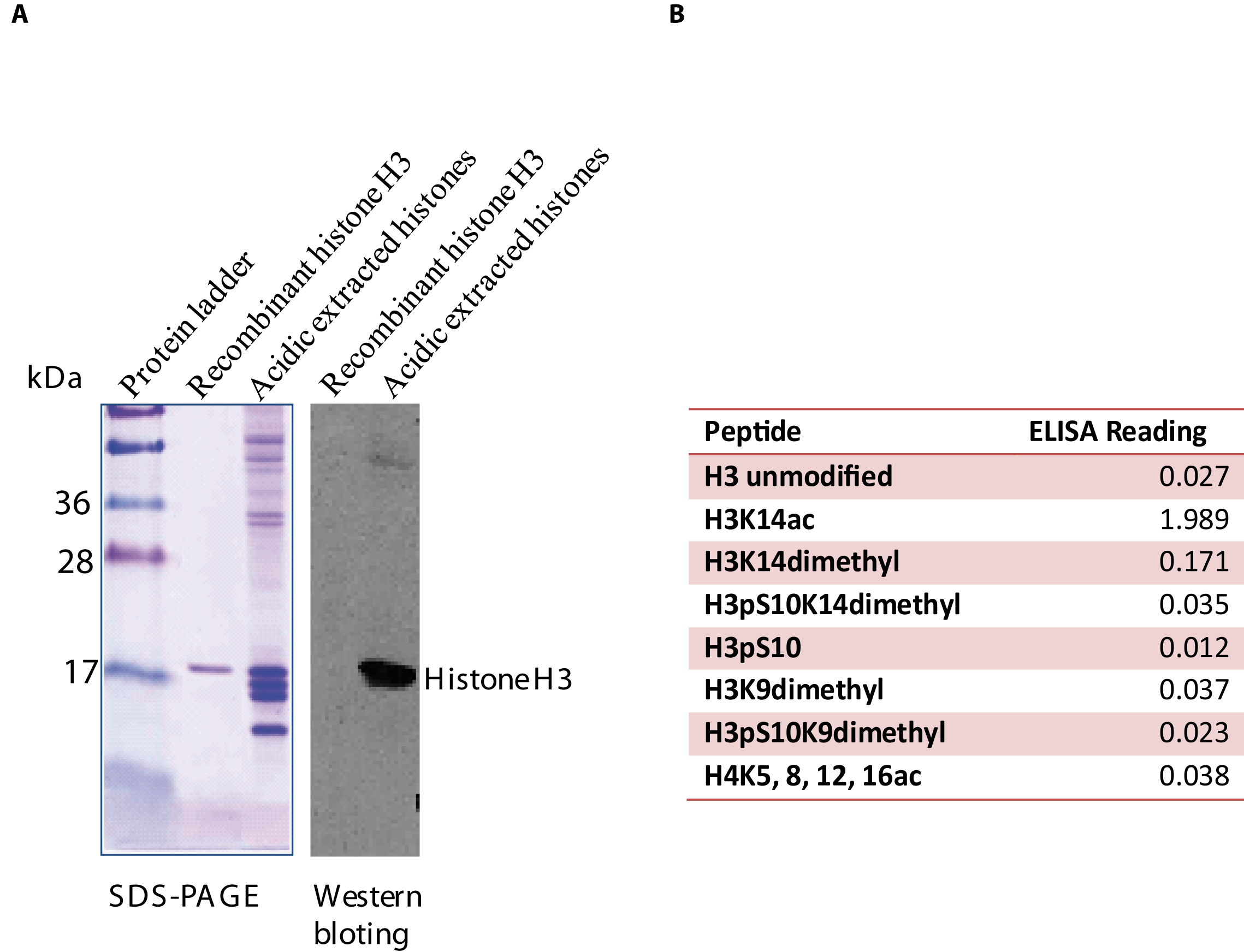
**
