# Supplementary material for: H3K9 and H3K14 acetylation co-occur at many gene regulatory elements, while H3K14ac marks a subset of inactive inducible promoters in mouse embryonic stem cells
Source: BMC Genomics. 2012 Aug 24;13:424. doi: 10.1186/1471-2164-13-424 (PMC3473242; doi:10.1186/1471-2164-13-424)
Supplement: Additional file 3 — Figure S3. Validation of (A) H3K9ac and (B) H3K14ac ChIP-seq in mouse embryonic stem cells by ChIP-qPCR. Peaks of local enrichment of H3K9 and H3K14 acetylation were determined after sequence alignment and normalization to input DNA. Tag density per peak (shown on the right of the graph) is plotted as fold enrichment over an arbitrarily chosen control genomic region. The list of primer is provided as Supplementary Table S1. These ChIP-qPCR results confirmed the specificity of the predicted peaks for H3K9 and H3K14 acetylation. [file 1471-2164-13-424-S3.doc]

**Additional File 3: Supplementary Figure S3. Validation of (A) H3K9ac and (B) H3K14ac ChIP-seq in mouse embryonic stem cells by ChIP-qPCR.** Peaks of local enrichment of H3K9 and H3K14 acetylation were determined after sequence alignment and normalization to input DNA. Tag density per peak (shown on the right of the graph) is plotted as fold enrichment over an arbitrarily chosen control genomic region. The list of primer is provided as Supplementary Table S1. These ChIP-qPCR results confirmed the specificity of the predicted peaks for H3K9 and H3K14 acetylation.

**
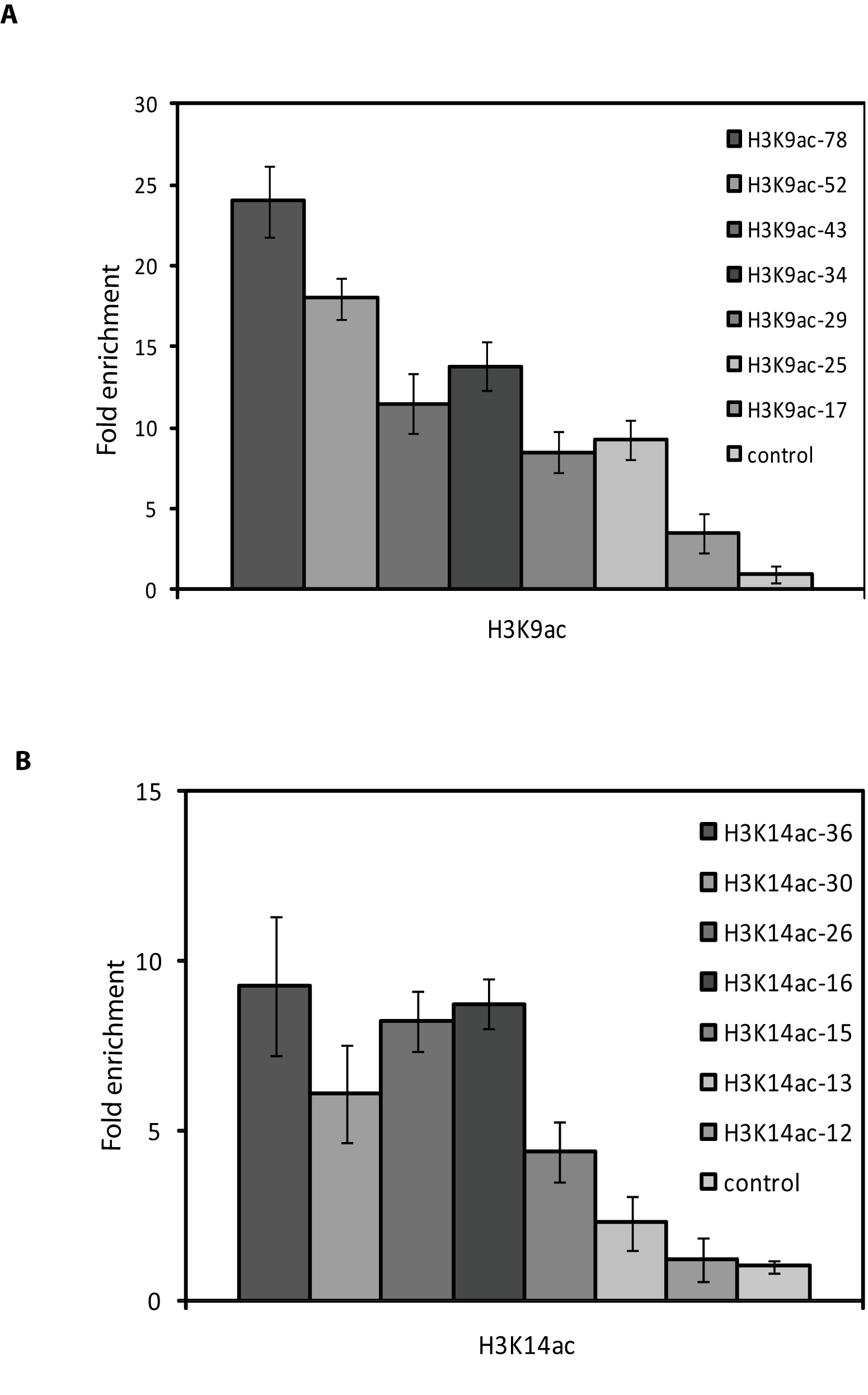
**
