# Supplementary material for: H3K9 and H3K14 acetylation co-occur at many gene regulatory elements, while H3K14ac marks a subset of inactive inducible promoters in mouse embryonic stem cells
Source: BMC Genomics. 2012 Aug 24;13:424. doi: 10.1186/1471-2164-13-424 (PMC3473242; doi:10.1186/1471-2164-13-424)
Supplement: Additional file 4 — Table S1. List of primers used for ChIP-qPCR validation. [file 1471-2164-13-424-S4.doc]

**Additional File 4: Supplementary Table S1. List of primers used for ChIP-qPCR validation.**

**Primers used in Figure 1E**

| Primers | | Forward primer | | Reverse primer | | |
| --- | --- | --- | --- | --- | --- | --- |
| Rnf25 |  | | GGGAGCAATGGGGTCGATAA | | TCAAGGGGCGAGGGATAGAA |  |
| Hspd1 |  | | CCCGAGCAAGGTCAAAGTGAA | | GGCTAGGGTGGAAGCAACTT |  |
| B230219D22Rik | | | ACGGCCTCCCACACTTGTTT | | AAGCTTCAAAGGCCCACACG |  |
| Hnrnpa2b1 |  | | CAGTCGCTTCAGCCCGATTT | | CTTTGCGGCCCAGTTTCTTG |  |
|  | | |  | |  |  |
|  |  | |  | |  |  |
|  |  | |  | |  |  |

**Primers used in Figure 5B and 5C.**

| Sntg1 | |  | | | TTGTGCAGCGCAGACTTGTG | | ACGCTCGTGCAATCACTCCA | |  | | | |
| --- | --- | --- | --- | --- | --- | --- | --- | --- | --- | --- | --- | --- |
| TCFAP2D | |  | | | GCGCCTTGGTTGCAAAATG | | GGCAACCGTGGAATTGGCTA | |  | | | |
| Gabra4 | |  | | | CAAGCTTGCAAGTGCTGCTCA | | TTGGGGTTTTGAAGTCGCTGA | |  | | | |
| GRM8A | |  | | | TGGCTGTCAGCTGCATGGTT | | GCAAAAGAAAGCCCCCACCT | |  | | | |
| Hhip | |  | | | TGTTCTCCAGACGCCCCAAT | | CCCCAAAGCGCCTAAAGAGAA | |  | | | |
|  | |  | | |  | |  | |  | | | |
|  | **Primers used in Figure 7** | | | | | | | | |  | |  |
|  | Hspd1 | | |  | | CCCGAGCAAGGTCAAAGTGAA | | GGCTAGGGTGGAAGCAACTT | | |  | |
|  | B230219D22Rik | | | | | ACGGCCTCCCACACTTGTTT | | AAGCTTCAAAGGCCCACACG | | |  | |
|  | 4930486L24Rik | | | | | TGGGAACCAGTGCAGAGTTGAG | | AAAGCAGGCAGATCCCTCAGTG | | |  | |
|  | Dsc1 | |  | | | CCCTGCCTTCGATTCTCTCAAG | | ACTTCAGATGGCTGTGGCCTGT | | |  | |

**Primers used in Supplementary Figure S3.**

|  |  |  |
| --- | --- | --- |
| H3K9ac-78 | CCTCCCACAACAAAACAACC | GTCGATGGAACCAGCCAATA |
| H3K9ac-58 | CCCGAGCAAGGTCAAAGTGAA | GGCTAGGGTGGAAGCAACTT |
| H3K9ac-41 | TAGCAGATGTGGGTGGGTTGTG | ACACGATGGAGGAGGAGTTGGA |
| H3K9ac-30 | ACACCCACTTGGTAATCACAAACA | CCAATCAAGGAACAGAGGATGTCA |
| H3K9ac-27 | CTCTCACTTGTCGCCATTCTGC | CGATGAGAGCATCAGAACATTTCC |
| H3K9ac-25 | GGGAGCAATGGGGTCGATAA | TCAAGGGGCGAGGGATAGAA |
| H3K9ac-15 | TGAATGAAAAGCCACAGACCACTC | TCAATTTGGCTGGCTCACCTT |
| control | TCTGCCCAGTTTCTGGTTTGC | ATGCGCAAAGCCAAGGTCTC |
|  |  |  |
|  |  |  |
| H3K14ac-36 | GGACTGTATTCCGAGGGAGAGGTT | CAACATGCGAACACGGAGGA |
| H3K14ac-30 | CAGTCGCTTCAGCCCGATTT | CTTTGCGGCCCAGTTTCTTG |
| H3K14ac-26 | CAGTCGCTTCAGCCCGATTT | CTTTGCGGCCCAGTTTCTTG |
| H3K14ac-16 | ACGGCCTCCCACACTTGTTT | AAGCTTCAAAGGCCCACACG |
| H3K14ac-15 | GCTTTGCTGGCTTCTGATGGA | AAAAGCGGAAGCCCTTGGAC |
| H3K14ac-13 | CCCGGCATTTGCGTCTCTAT | AAGGCTCGTCCCCAAAATGC |
| H3K14ac-12 | GTGCTGCCACACCCAAAAAG | TTGGCCTTGGCTGGACTCTT |
| control | TCTGCCCAGTTTCTGGTTTGC | ATGCGCAAAGCCAAGGTCTC |
