# Supplementary material for: H3K9 and H3K14 acetylation co-occur at many gene regulatory elements, while H3K14ac marks a subset of inactive inducible promoters in mouse embryonic stem cells
Source: BMC Genomics. 2012 Aug 24;13:424. doi: 10.1186/1471-2164-13-424 (PMC3473242; doi:10.1186/1471-2164-13-424)
Supplement: Additional file 5 — Figure S4. Enrichment of H3K9ac and H3K14ac over various genomic regions. Average enrichment profile of H3K9 and H3K14ac over the promoters, coding exons, introns and distal intergenic regions. Enrichment over distal intergenic and intronic sites is comparable to promoters. [file 1471-2164-13-424-S5.doc]

**Additional File 5: Supplementary Figure S4. Enrichment of H3K9ac and H3K14ac over various genomic regions.** Average enrichment (tag density) profile of (A) H3K9 and (B) H3K14ac over the promoters, coding exons, introns and distal intergenic regions. Enrichment over distal intergenic and intronic sites is comparable to promoters. Y-axis represents enrichment of mean tag density of H3K9ac and H3K14ac signals at the different genomic locations tested.

**
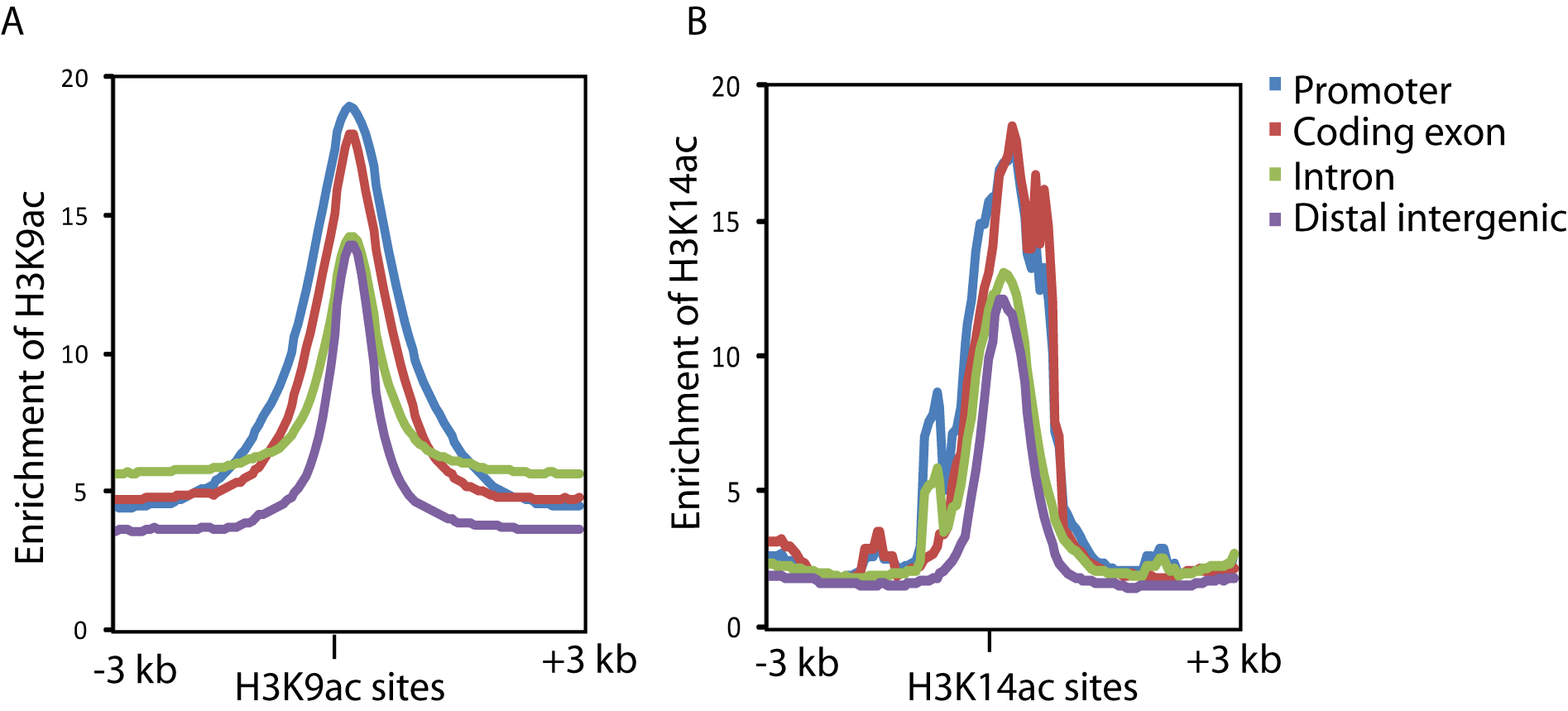
**
