# Supplementary material for: H3K9 and H3K14 acetylation co-occur at many gene regulatory elements, while H3K14ac marks a subset of inactive inducible promoters in mouse embryonic stem cells
Source: BMC Genomics. 2012 Aug 24;13:424. doi: 10.1186/1471-2164-13-424 (PMC3473242; doi:10.1186/1471-2164-13-424)
Supplement: Additional file 6 — Figure S5. Correlation of H3K9ac (A) and H3K14ac (B) intergenic peaks with H3K4me1, H3K27ac, Pol II and p300. Heatmaps of the signal density using k-means clustering over H3K9ac and H3K14ac distal intergenic sites (-/+ 5 kb). Strong correlation with H3K4me1 and H3K27ac and presence of Pol II and p300 over H3K9ac and H3K14ac intergenic sites suggest that they may act as enhancers. [file 1471-2164-13-424-S6.doc]

**Additional File 6: Supplementary Figure S5. Correlation of H3K9ac (A) and H3K14ac (B) intergenic peaks with H3K4me1, H3K27ac, Pol II and p300.** Heatmaps of the signal density using k-means clustering over H3K9ac and H3K14ac distal intergenic sites (-/+ 5 kb). Strong correlation with H3K4me1 and H3K27ac and presence of Pol II and p300 over H3K9ac and H3K14ac intergenic sites suggest that they may act as enhancers.

**
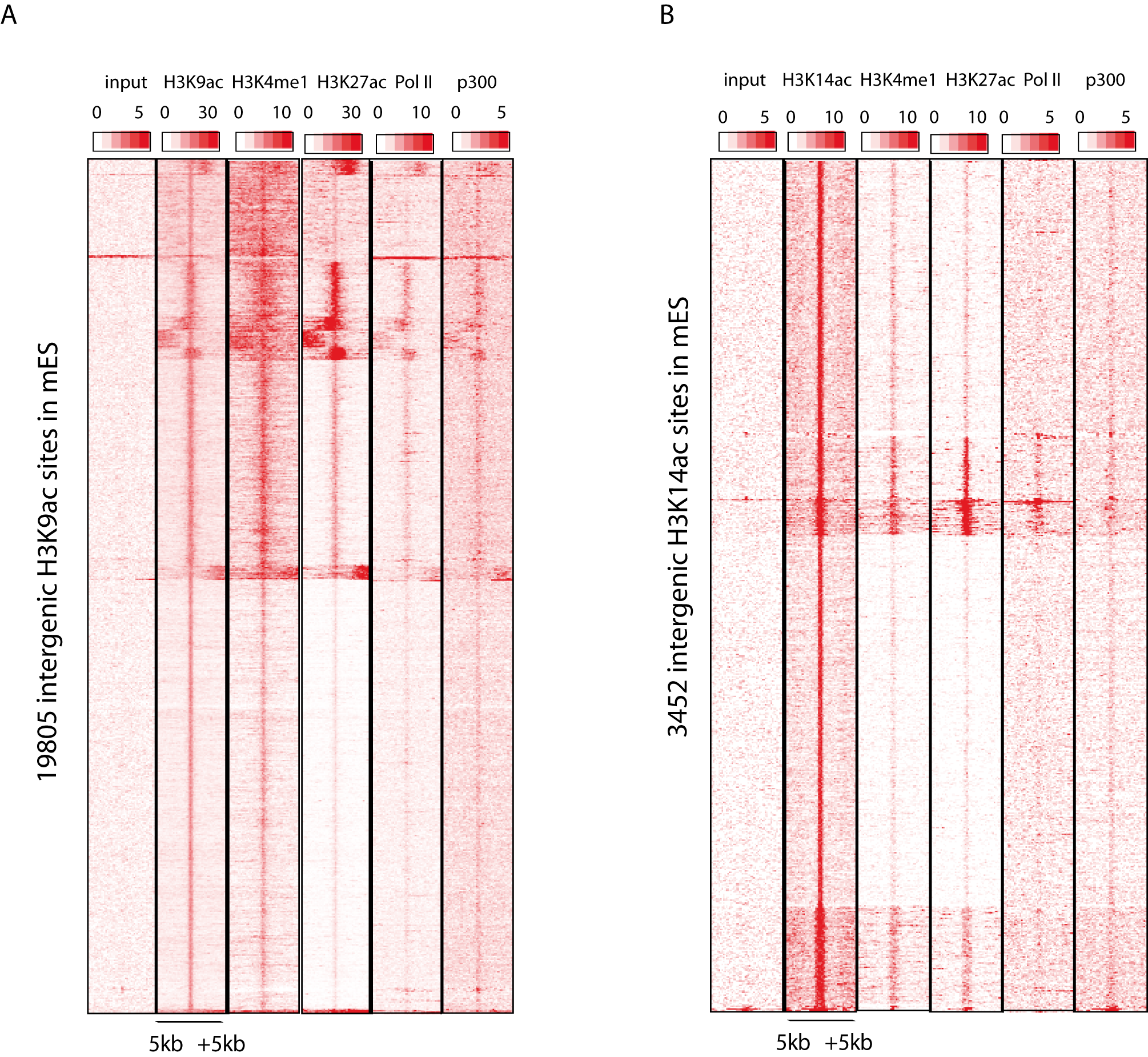
**
