# Supplementary material for: H3K9 and H3K14 acetylation co-occur at many gene regulatory elements, while H3K14ac marks a subset of inactive inducible promoters in mouse embryonic stem cells
Source: BMC Genomics. 2012 Aug 24;13:424. doi: 10.1186/1471-2164-13-424 (PMC3473242; doi:10.1186/1471-2164-13-424)
Supplement: Additional file 7 — Figure S6. Measurement of Oct4 level at various time points after the treatment with 5 mg/ml sodium butyrate in ES cells. Level of Oct4 was monitored by Western blot to see the pluripotent state of the ES cells at various time points after sodium butyrate treatment. The similar levels of Oct4 observed at different experimental time points suggest that the time points we have used for sodium butyrate treatment have no effect on pluripotency of the ES cells. Tubulin was used as a loading control. [file 1471-2164-13-424-S7.doc]

**Additional File 7: Supplementary Figure S6. Measurement of Oct4 level at various time points after the treatment with 5mg/ml sodium butyrate in ES cells.** Level of Oct4 was monitored by Western blot to see the pluripotent state of the ES cells at various time points after sodium butyrate treatment. Similar level of Oct4 at experimental time points suggest that the time points we have used for sodium butyrate treatment have no effect on pluripotency of the ES cells. Tubulin was used as a loading control.

**
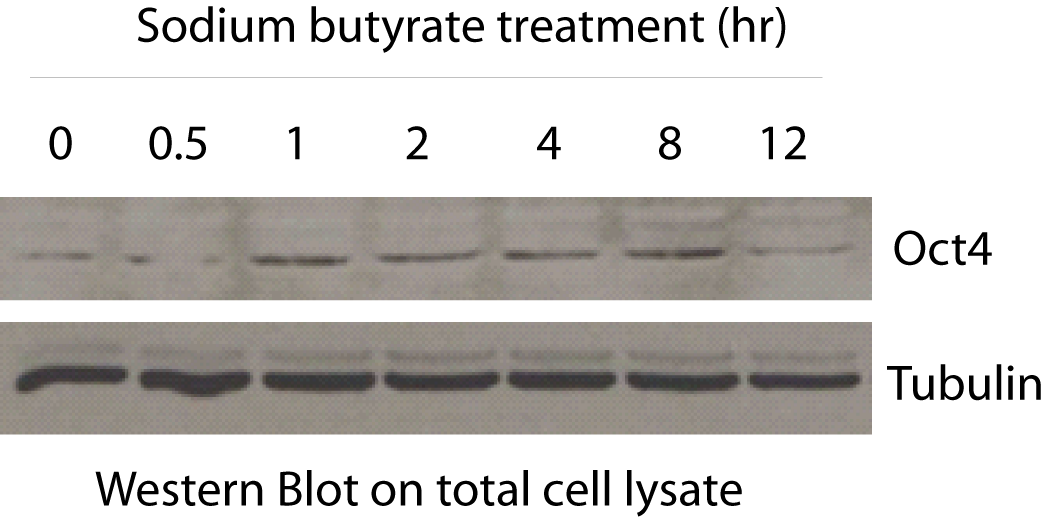
**
