# Supplementary material for: H3K9 and H3K14 acetylation co-occur at many gene regulatory elements, while H3K14ac marks a subset of inactive inducible promoters in mouse embryonic stem cells
Source: BMC Genomics. 2012 Aug 24;13:424. doi: 10.1186/1471-2164-13-424 (PMC3473242; doi:10.1186/1471-2164-13-424)
Supplement: Additional file 8 — Figure S7. Ratio of tag density of least expressed to highest expressed gene promoters for H3K9 and H3K14 acetylation. H3K9 and H3K14 acetylation tag density were calculated over 500 least and highest expressed gene promoters based upon their RNA-sequencing expression profile. The ratio of tag density of least expressed to highest expressed plotted for H3K9 and H3K14 acetylation, which shows that H3K14ac is specifically enriched at inactive promoters as compared to H3K9ac. [file 1471-2164-13-424-S8.doc]

**Additional File 8: Supplementary Figure S7. Ratio of tag density of least expressed to highest expressed gene promoters for H3K9 and H3K14 acetylation.** H3K9 and H3K14 acetylation tag density were calculated over 500 least and highest expressed gene promoters based upon their RNA-sequencing expression profile. The ratio of tag density of least expressed to highest expressed plotted for H3K9 and H3K14 acetylation, which shows that H3K14ac is specifically enriched at inactive promoters as compared to H3K9ac.

**
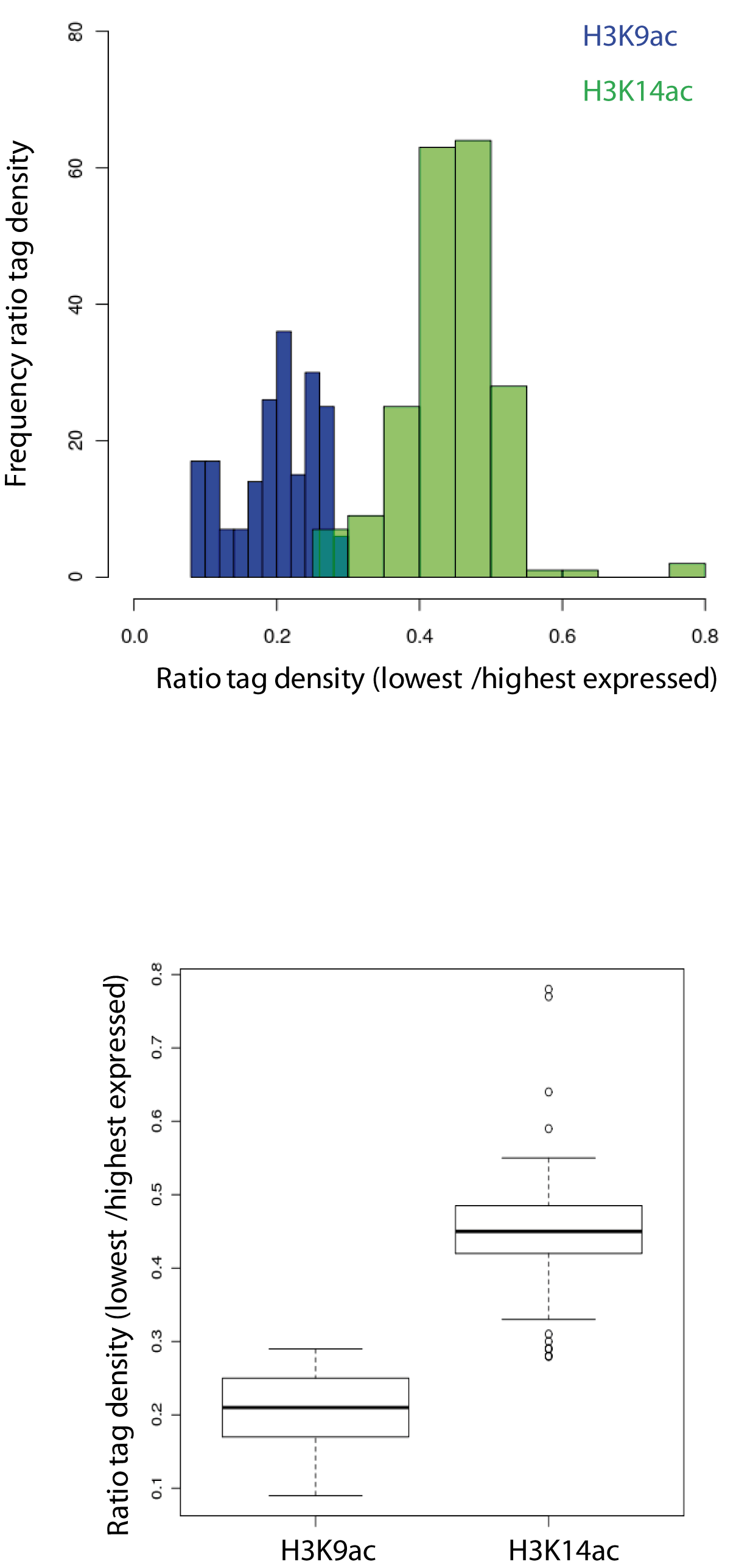
**

**
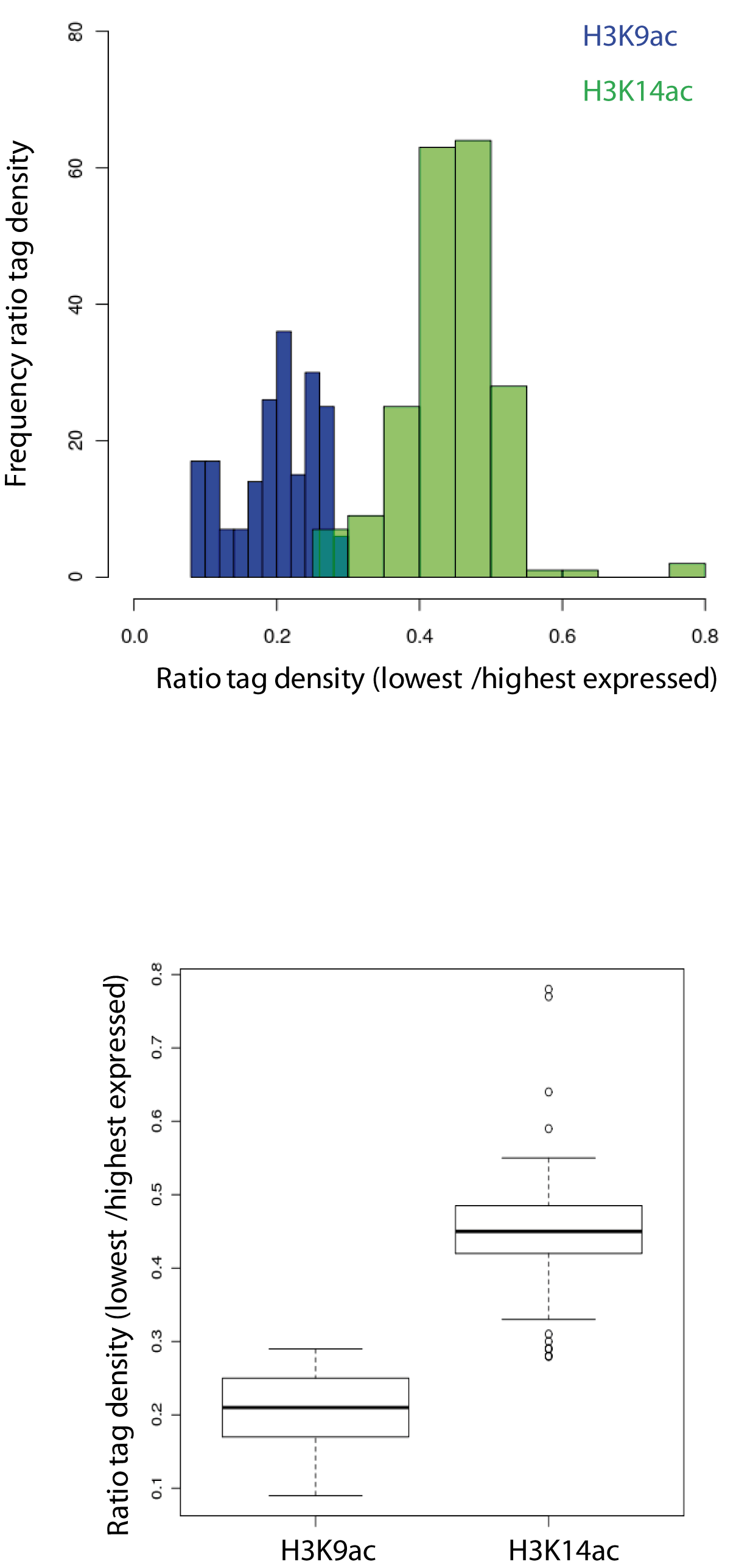
**
