# Supplementary material for: H3K9 and H3K14 acetylation co-occur at many gene regulatory elements, while H3K14ac marks a subset of inactive inducible promoters in mouse embryonic stem cells
Source: BMC Genomics. 2012 Aug 24;13:424. doi: 10.1186/1471-2164-13-424 (PMC3473242; doi:10.1186/1471-2164-13-424)
Supplement: Additional file 9 — Table S2.Number of mapped reads for the data set used in this study. [file 1471-2164-13-424-S9.doc]

**Additional File 9: Supplementary Table S2**. Number of mapped reads for the data set used in this study.

| **Sample** | **Cell type** | **Aligned Reads** | **Mapped peaks** | **Accession ID** |
| --- | --- | --- | --- | --- |
| **H3K9ac** | mES | 2.31E+007 | 60204 | GSM775313 |
| **H3K14ac** | mES | 2.23E+007 | 8105 | GSM775314 |
| **H3K27ac** | mES | 1.14E+007 | NA | GSM594578 |
| **H3K4me1** | mES | 8.20E+006 | NA | GSM594577 |
| **H3K4me3** | mES | 8.84E+006 | NA | GSM307618 |
| **H3K9me3** | mES | 4.23E+006 | NA | GSM307621 |
| **H3K27me3** | mES | 6.53E+006 | NA | GSM307619 |
| **Pol II** | mES | 2.68E+006 | NA | GSM307623 |
| **Total H3** | mES | 4.39E+006 | NA | GSM307624 |
| **Input** | mES | 2.73E+006 | NA | GSM307625 |
| **p300** | mES | 8.22E+006 | NA | GSM699164 |
| **RNA-seq** | mES | - | NA | GSM632013 |
